# Dimensions of pathological narcissism and intention to vote for Donald Trump

**DOI:** 10.1371/journal.pone.0249892

**Published:** 2021-04-15

**Authors:** Matthew M. Yalch

**Affiliations:** Palo Alto University, Palo Alto, CA, United States of America; Univeristy of Padova, ITALY

## Abstract

Pathological narcissism is a term often applied to former President Donald Trump, but it has been less examined as a potential predictor of voting for him. Trump projects a grandiose and omnipotent self-image during press conferences and rallies, and his followers at these events often respond with both effusive admiration and an inflated sense of their own self-regard, all of which are aspects of narcissism. However, while Trump’s personal narcissism has been well documented, there is little research on the narcissism of his supporters. In this study we conducted an exploratory analysis examining the hierarchical structure of pathological narcissism and which aspects of narcissism within that structure were associated with intended voting for Trump in the 2020 U.S. presidential election in a sample of U.S. residents collected online (*N* = 495) using Amazon’s Mechanical Turk. Results indicated that an eight-echelon hierarchy best fit the data. Within this hierarchy, antagonistic and indifferent aspects of narcissism within the fifth echelon best predicted intended voting for Trump over and above relevant demographic variables. These results have implications for the study of narcissism and, especially given the results of the 2020 election, the degree to which one can make use of narcissistic aspects of personality in political contests.

## Introduction

Pathological narcissism is a term often applied to former President Donald Trump [1]. Less commented upon is what leads people to vote for him. Research suggests that demographic factors influence voting for Trump (e.g., people who are White, male, older, and less educated are more likely to vote for him), but identity politics explains only a portion of people’s voting behavior [2, 3]. There is reason to suspect that Trump’s narcissism may reflect in some ways the narcissism of those who vote for him. However, there is little research on the association between narcissism and likelihood of voting for Donald Trump.

## Dynamics and structure of pathological narcissism

Narcissism has a long and ambiguous history (for review, see [4]). In diagnostic and colloquial terms, narcissism is often understood in terms of grandiosity, inflated self-esteem, and feeling entitled and superior to others [5–7]. However, perhaps a more accurate and clinically informed view is that narcissism is a *process*. In this process, the grandiosity characteristic of narcissism functions as a defense against feelings of vulnerability, inferiority, and generally not being good enough [5, 6, 8–10]. Although some have argued that narcissism is a normal aspect of development (e.g., [9]), it becomes pathological when it causes disruption in the lives of the narcissistic person and/or those around them.

Narcissistic grandiosity and vulnerability are multifaceted dimensions that manifest in different ways [5, 6, 11]. Grandiosity varies in degrees of severity from excessive and unwarranted self-esteem to delusions of grandeur; it varies in form from arrogance and entitlement to gratuitous need for attention to Machiavellian exploitation of and willful aggression towards others reminiscent of psychopathy [11, 12]. These latter, sadistic variants of grandiosity entail a lack of empathy for other people and may be referred as *malignant* narcissism [12, 13]. Vulnerability may manifest as hypersensitivity to criticism, emotional lability, masochistic tendency to sacrifice oneself, and/or affinity for and admiration of others of higher status.

Research on these different aspects of narcissism suggests a hierarchical structure of pathological narcissism, with overall narcissistic pathology constituting the top of the hierarchy. This differentiates into dimensions of grandiosity and vulnerability, which in turn split into a trifurcate structure of grandiosity, vulnerability, and an entitled, self-centered antagonism factor at the third echelon of the hierarchy. These different dimensions of narcissism continue to divide within the structural hierarchy, with distrust emerging at the fourth echelon, attention-seeking at the fifth, and so on [14–18]. These dimensions manifest in varying ways and degrees of severity across different people.

### Narcissism, Donald Trump, and voting

A number of psychologists and other mental health experts have suggested that Donald Trump appears pathologically and malignantly narcissistic (e.g., [19–23]; see also [24]). To these experts, his grandiosity is fairly straightforward: he has stated innumerable times in many public forums that he is a “stable genius”, knowing more than anyone else about a wide variety of topics (e.g., technology, infrastructure, the economy; for review see [25, 26]). His statements are often self-centered and show little regard for other people, and he lies effortlessly and without compunction [26, 27]. Biographical analyses of Donald Trump have suggested that his grandiosity may belie a vulnerability to feeling weak and unimportant, and consequently he is very sensitive to being wrong and needs constant reassurance of his greatness and importance [21, 22].

Many have argued that one result of Donald Trump’s narcissism and the behaviors following from it is that his presidency has been rocky (for journalistic chronicles of the Trump presidency, see [26–28]; for more candid critiques from within the Republican Party see [25, 29]). For example, Trump and those in his administration have been embroiled in legal disputes since the beginning of his term (which contributed to an unusually high degree of staff turnover), many of his domestic and international policies were unsuccessful, and due to his handling of the COVID-19 pandemic, over 400,000 Americans died of the virus over the course of his presidency [26–28, 30]. Consequently, Trump’s approval rating in the U.S. never approached 50% and he was impeached by Congress approximately mid-way through his term and again at the end of his term [31].

Although most Americans did not approve of Donald Trump and his performance as president, there is a large and vocal minority who very much did and do [32]. To the extent that Trump has a cohesive political platform, it focuses on appealing to people who may identify with and thus voted for him: heterosexual, older White men without a college education (e.g., [3]). This appeal extends more broadly to regions in which potential supporters may cluster (i.e., states in which the Republican Party predominates). However, more than just identity influences voting [2]. The pathological narcissism that some have attributed to Donald Trump repels many people who are secure in their identity, but it may attract those who may be less secure, who may think of themselves as exceptional but feel undervalued and who thus identify with the grandiose and aggressive aspects of Donald Trump, using this identification to defend against their own vulnerability [13, 32, 33]. In effect, Trump’s narcissism may attract the support of those who also have narcissistic tendencies (and, additionally, may reinforce these tendencies). Indeed, at Trump’s rallies and other public events, his followers often respond with what might be considered effusive admiration and an inflated sense of their own self-regard, manifesting aspects of both narcissistic grandiosity and vulnerability. It is unclear the degree to which one or both dimensions of narcissism might influence how people actually vote. Research suggests that narcissistic entitlement (an aspect of narcissistic grandiosity) is associated with political conservatism in general [34]. Research further suggests that collective narcissism (the feeling one’s in-group is exceptional, which is linked to narcissistic vulnerability) is associated with voting for Trump for president in 2016 in particular [32] as well as with holding a positive opinion of him post-election [35]. However, there is little research examining what aspects of pathological narcissism might have predicted voting for Trump for re-election in 2020.

## Current study

In this study we examined the influence of pathological narcissism on intended voting for Donald Trump for President of the United States in 2020. Although we expected that aspects of both narcissistic grandiosity and narcissistic vulnerability would increase the likelihood of intending to vote for Trump over and above other demographic features, we did not have explicit *a priori* hypotheses about which specific facets of grandiose and/or vulnerable narcissism might predict intended vote for him, at which echelon within the hierarchical structure of pathological narcissism these facets might be, or how specifically this structure might unfold. Because of this, the analyses of this study were primarily exploratory.

## Method

### Participants

Participants in this study were 779 men and women living in the U.S. employed as workers on Amazon’s Mechanical Turk (Mturk). Although Mturk is in general a source of valid data for the social and behavioral sciences (for review see [36]), we nonetheless took several steps to ensure that the responses participants provided were of high quality. First, we required survey respondents to complete a Captcha to ensure they were not bots. Second, we automatically directed survey respondents out of the survey without compensation if they provided incorrect answers on any of three items (“Which of the following is cold?”, “Which of the following would a person wear on their head?”, and “5 + 1 =“). Third, of those respondents who made it to the end of the survey, we deleted responses from those who did not respond to 10% or more of survey items (*n* = 197). Finally, we removed from analysis those who scored at or above 84*T* on the Infrequency scale of the *Personality Assessment Inventory* [37], a scale designed to detect random or unusual responding (*n* = 87). This yielded a final sample of 495 people, who took on average 15.56 minutes (933.88 seconds, *SD* = 1087.68, range: 85–19,394) to complete the survey.

Participants were approximately equal numbers of men and women, had a mean age of 41.81 years (*SD* = 12.73, range: 18–79), and were majority White, heterosexual, and college-educated (see Table 1). The majority of participants were registered voters and intended to vote for Joe Biden, although they were more heterogeneous with respect to political party identification. They represented 45 of 50 states (plus the District of Columbia [DC]) with number of participants per state ranging from 57 (12%, California) to 1 (< 1%, Hawaii, New Mexico, Wyoming, DC) with an average of 11 people (2%) per state (for details see S1 Table in S1 File). After providing informed consent for the study, participants completed an online survey in exchange for payment. All survey procedures received approval from the Palo Alto University Institutional Review Board (application #2020–109) and study data are available on Open Science Framework (https://osf.io/sedyp/).

**Table 1:** Demographic and political preferences of participants.

	*n* (%)
Sex	
Male	263 (53%)
Female	228 (46%)
Other	2 (< 1%)
Sexual orientation	
Heterosexual	420 (85%)
Gay/Lesbian	15 (3%)
Bisexual	55 (11%)
Other	5 (1%)
Race	
Asian	26 (5%)
Black	49 (10%)
Native American	11 (2%)
White	390 (79%)
Multiracial	8 (2%)
Other	6 (1%)
LatinX	47 (9%)
Education	
Some schooling	2 (< 1%)
High school/GED	49 (10%)
Some college	100 (20%)
Bachelor’s degree	252 (51%)
Master’s degree	83 (16%)
Doctorate	8 (2%)
Registered voter	463 (94%)
Political party	
Democratic	217 (44%)
Republican	170 (34%)
Libertarian	5 (1%)
Socialist	7 (1%)
Independent	86 (17%)
Other	10 (2%)
Intended vote in 2020 presidential election	
Donald Trump	201 (41%)
Joe Biden	253 (51%)
Other/I don’t know	41 (8%)

### Measures

Participants completed the following scales in the order listed, after which they answered they answered questions about political affiliation, voting, and demographics, including the dependent variable in the study “Which candidate do you plan to vote for in the 2020 U.S. presidential election”, which had three possible answers (Donald Trump, Joe Biden, and Other/I don’t know). Since the purpose of this study was voting for Donald Trump specifically, we dichotomized this question such that score of 1 indicated voting for Trump and a score of 0 indicated endorsement of either of the other options. We calculated all scales by taking the mean rating of items within each scale.

#### Five Factor Narcissism Inventory Short Form (FFNI-SF)

The FFNI-SF [38] is a 60-item survey measuring 15 facets of grandiose and vulnerable narcissism using items drawn from the Five-Factor Model of personality [39, 40]. Participants rated how much they agreed with each item on a 5-point scale ranging from “disagree strongly” to “agree strongly.” The FFNI contains 15 subscales: Acclaim-Seeking (α = .90; example item: “I have a tremendous drive to succeed”), Arrogance (α = .88; example item: “I only associate with people of my caliber”), Authortativeness (α = .91; example item: “leadership comes easy for me”), Distrust (α = .42; example item: “I often think that others aren’t telling me the whole truth”), Entitlement (α = .92; example item: “I deserve to receive special treatment”), Exhibitionism (α = .84; example item: “I like being the most popular person at a party”), Exploitativeness (α = .93; example item: “sometimes to succeed you need to use other people”), Grandiose Fantasies (α = .77; example item: “someday I believe that most people will know my name”), Indifference (α = .87; example item: “I don’t really care what others think of me”), Lack of Empathy (α = .90; example item: “I don’t get upset by the suffering of others”), Manipulativeness (α = .90; example item: “I can talk my way into and out of anything”), Need for Admiration (α = .74; example item: “I wish I didn’t care so much about what others think of me”), Reactive Anger (α = .86; example item: “it really makes me angry when I don’t get what I deserve”), Shame (α = .85; example item: “I feel foolish when I make a mistake in front of others”), and Thrill-Seeking (α = .93; example item: “I am a bit of a daredevil”). The internal consistency of one of the facets (Distrust) is lower than what is typically considered acceptable. However, since the FFNI (and other scales of personality pathology like it based on the FFM) are composites made of different aspects of the FFM, alpha may not be the most appropriate measure of internal consistency (for review of FFM-based measures of personality pathology see [41]).

#### Pathological Narcissism Inventory (PNI)

The PNI [42] is a 52-item survey measuring seven pathological dimensions of grandiose and vulnerable narcissism. Participants rated how much each item resembled them on a 6-point scale ranging from “not at all like me” to “very much like me.” The PNI contains 7 subscales: Contingent Self-Esteem (α = .96; example item: “when others don’t notice me, I start to feel worthless”), Exploitative (α = .88; example item: “I can make anyone believe anything I want them to”), Self-Sacrificing Self-Enhancement (α = .83; example item: “I like to have friends who rely on me because it makes me feel important”), Hiding the Self (α = .82; example item: “I can’t stand relying on other people because it makes me feel weak”), Grandiose Fantasy (α = .93; example item: “I want to amount to something in the eyes of the world”), Devaluing (α = .92; example item: “Sometimes I avoid people because I’m concerned that they’ll disappoint me”), and Entitlement Rage (α = .93; example item: “It irritates me when people don’t notice how good a person I am”).

#### Narcissistic Admiration and Rivalry Questionnaire (NARQ)

The NARQ [43] is an 18-item survey measuring the degree to which people expect admiration and view themselves competitively with respect to others across six facets. Participants rated how much they agreed with each item on a 6-point scale ranging from “not agree to all” to “agree completely.” The NARQ contains 6 subscales: Grandiosity (α = .85; example item: “I am great”), Uniqueness (α = .71; example item: “being a very special person gives me a lot of strength”), Charmingness (α = .78; example item: “mostly, I am very adept at dealing with other people”), Devaluation (α = .85; example item: “most people won’t achieve anything”), Supremacy (α = .90; example item: “I secretly take pleasure in the failure of my rivals”), and Aggressiveness (α = .82; example item: “I often get annoyed when I am criticized”).

#### Hypersensitive Narcissism Scale (HSNS)

The HSNS [44] (α = .83) is a 10-item scale measuring narcissistic hypersensitivity. Participants rated how much each item (example: “I often interpret the remarks of others in a personal way”) resembled them on a 6-point scale ranging from “very uncharacteristic or untrue, strongly disagree” to “very characteristic or untrue, strongly agree.”

### Procedures

The survey was available on Mturk under the name “Personality and Politics Study” from October 7–19, 2020, towards the beginning of the COVID-19 pandemic during which time much of the country was in quarantine. Workers were eligible to participate in the study if they were residents of the U.S. and completed previous assignments on Mturk (Human Intelligence Tasks [HITs]) with a 99% approval. Workers received $1 USD compensation upon completion. During the time that the survey was available several potentially relevant events took place. Just prior to the survey, there were 7.53 million confirmed cases of and over 211,000 deaths from COVID-19. Donald Trump himself contracted COVID-19 and returned from the hospital against medical advice, after which he made a speech in which he stated that this disease was not a major concern, and later stated that he would reject a COVID-related economic stimulus package. Following this, there was a 600-point swing (down 1.3%) in the Dow-Jones Index (down 1.4% in S&P, 1.6% in Nasdaq) [45]. Twenty-three members of Trump’s White House staff and others in his orbit also tested positive for COVID-19 [46]. Also during this time, unemployment was 7.9% [47], Amy Coney Barrett had been nominated (but not yet confirmed) to the U.S. Supreme Court, and the first presidential debate aired [48].

### Data analysis

We examined the hierarchical structure of pathological narcissism using a hierarchical approach to factor analysis [49]. Specifically, we entered 29 different subscales of narcissism into a principal components analysis (orthogonal Varimax rotation), extracting one component, then two, then three, and so on until no subscale had its highest loading on a new factor. Following this, we correlated each factor solution with the one above and below it to construct a structural hierarchy in which factors within each echelon were uncorrelated with each other but selectively correlated with factors at echelons above and below it. This approach is useful in that it provides information about how factor structural unfolds. An additional benefit is that the factors within each structure can serve as predictors in regression models with no concerns about multicollinearity since they were uncorrelated with each other.

We next entered orthogonal factor scores at each echelon of the structural hierarchy as predictors into a series of binomial logistic regression models in which intended vote for Trump for U.S. President in 2020 was the outcome variable and relevant demographic variables were covariates. For ease of interpretation, we recoded demographic variables such that 1 was a demographic aspect associated with voting for Donald Trump (e.g., male sex, White race, heterosexual orientation, affiliation with Republican Party, residence in a state that voted for Trump in 2016) and 0 was anything else (e.g., for sexual orientation, we coded gay/lesbian, bisexual, and other as 0). The exception to this was college degree status, for which we coded 1 with a degree and 0 without a degree. We constructed one regression model per echelon of the hierarchy plus one additional with only demographic variables (see [50]).

We estimated regression models within a Bayesian framework. In contrast to conventional approaches to data analysis, which evaluate observed effects with respect to a null hypothesis (expressed results in terms of *p*-values), Bayesian approaches to data analysis evaluate the relative probability of a range of possible effect estimates. Specifically, Bayesian models generate potential effect estimates via Markov chain Monte Carlo (MCMC) simulation and then compare these estimates with the data to establish their relative probability (for review see [51, 52]). Bayesian data analyses produce more accurate and informative estimates of effects than conventional data analyses [53] and because of this are increasingly used in psychological research [54] (for historical review see [55]). In this study we estimated each model using a low-information (flat) prior, thus allowing the data to have maximum influence on the results. Each model consisted of 3 chains of 20,000 iterations of Hamiltonian MCMC (HMC) simulation (and a burn-in period of 5,000 iterations), which we estimated using the *rstanarm* package in R [56]. We evaluated whether or not each chain of HMC simulation converged using Monte Carlo Standard Error and Potential Scale Reduction Factor diagnostics and through manual examination of autocorrelation and traceplots. All models terminated normally with no indication of non-convergence. We evaluated relative probability of models using the Leave-One-Out (LOO) cross-validation method, which compares two or more Bayesian models in terms of their relative probability in terms of the expected log pointwise predictive density (ELPD) statistic (for review see [57]). We conducted model LOO comparisons via the *loo* package in R [58].

## Results

Demographic variables had generally low correlations with voting for Trump with the exception of membership in the Republican Party, which had a large bivariate effect (*r* = .70). Facets of pathological narcissism had on average modest correlations with voting for Trump (*r*_μ_ = .19) and on average large correlations with each other (*r*_μ_ = .48). Correlations between pathological traits and demographic variables were generally modest (*r*_μ_ ≤ |.21|; for full correlation matrix see S2 Table in S1 File).

Although dichotomizing demographic features was more consistent with previous evidence and more expedient for our primary predictive analyses, it was also valuable to examine the association between intention to vote for Trump and demographic variables using more nuanced demographic categories. When we did this, we found that people endorsing Republican Party membership were more likely than members of every other political party (Democratic, Socialist, Independent, other) other than Libertarian to vote for Trump (χ^2^ = 253.02, *p* < .05; large effect: Φ = .71), although more Libertarians than not intended not to vote for Trump. People identifying as Latinx were also more likely to vote for Trump than people not identifying as Latinx (χ^2^ = 253.02, *p* < .05), but this effect was small (Φ = .10). People identifying as bisexual were more likely to vote for Trump than people identifying as gay (χ^2^ = 15.21), and both were equally (un)likely to vote for Trump than people identifying as heterosexual or other, although this effect was also small (Φ = .18). There were no differences in likelihood of voting for Trump based on biological sex (male, female, intersex, other; χ^2^ = 1.40, *ns*), gender (cisgender man, cisgender women, genderqueer, other; χ^2^ = 2.22, *ns*), or education status (some school, high school/GED, some college, bachelor’s degree, master’s degree, doctorate; χ^2^ = 5.06, *ns*; see S1 File for contingency tables).

**Fig 1:**
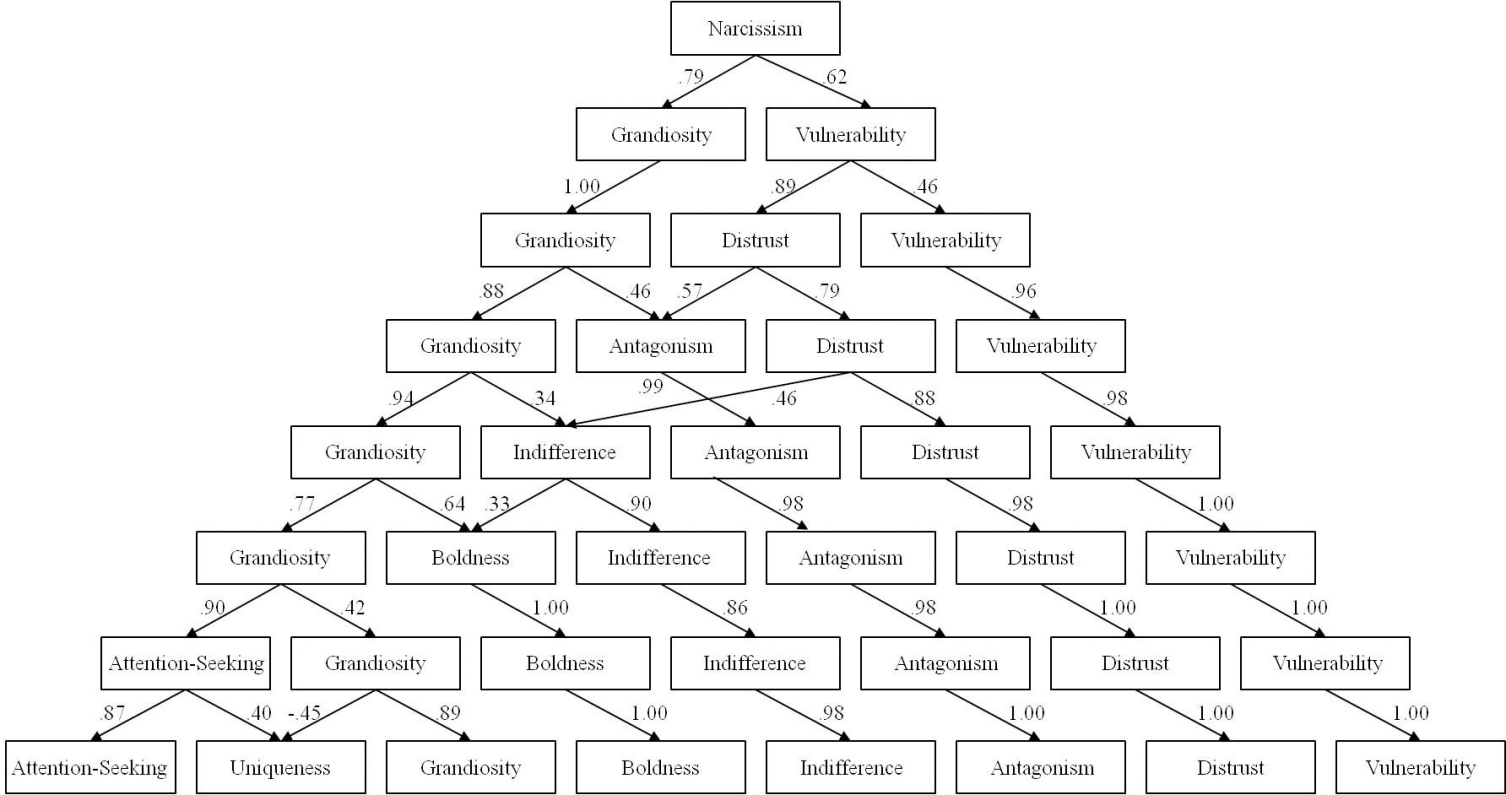


The hierarchical factor analysis yielded an eight-echelon structure of pathological narcissism (see Fig 1; for factor loadings at each echelon see S1 File). An overall factor of pathological narcissism was at the top of the hierarchy, which split into grandiosity and vulnerability in the second echelon. In the third echelon, vulnerability split to form a more specific vulnerability factor and an unempathic distrust factor. In the fourth, grandiosity split into a more specific grandiosity factor and a self-centered antagonism factor, to which distrust also contributed. In the fifth, a factor resembling indifference to other people emerged from grandiosity and distrust. In the sixth, a psychopathic boldness factor emerged from grandiosity and indifference. In the seventh, grandiosity split into an even more specific grandiosity factor and an attention-seeking factor, the latter of which split in the eighth echelon into a more specific attention-seeking factor and uniqueness factor.

We next entered factor scores from each echelon of the hierarchy into a series of binomial regression models predicting intended vote Trump in 2020. Since factors were uncorrelated at each echelon of the hierarchy, there was minimal multicollinearity in any of the regression models (across predictors in all models, VIF ≤ 1.32). Analysis of ELPD suggested that the model with fifth-echelon predictors was the most probable, although all models with narcissism were more likely than the model only demographic variables (ΔELPD ≥ 4.3). In this model, having a Bachelor’s degree or higher made people half as likely to vote for Trump, while being a member of the Republican Party made people over 48 times more likely to vote for Trump. Including narcissism as predictors accounted for a modest amount of additional variance (Δ*R*^2^ = .03), with higher levels of antagonism and indifference predicting a greater likelihood of intended voting for Trump (see Table 2; results of all models included in S1 File).

**Table 2:** Incremental effects of dimensions pathological narcissism on voting for Donald Trump in 2020.

	OR	B	95% HDI	*R*^2^
Age	1.00	.00	(-.02, .02)	.52*
Male	.83	-.19	(-.67, .28)	
White	1.45	.37	(-.24, 1.00)	
Hetero	1.35	.30	(-.38, 1.01)	
Degree	.60*	-.51	(-1.02, -.01)	
Trump-Voting State	.99	-.01	(-.47, .45)	
GOP	48.42*	3.88	(3.36, 4.42)	
Antagonism	1.93*	.66	(.41, .91)	
Grandiosity	1.13	.12	(-.12, .36)	
Vulnerability	1.23	.21	(-.02, .44)	
Distrust	.88	-.13	(-.36, .10)	
Indifference	1.43*	.36	(.12, .59)	

Note.

* indicates 0 is not within 95% HDI; HDI = highest density interval of parameter samples.

## Discussion

In this study we examined the hierarchical structure of pathological narcissism and the degree to which dimensions within each echelon of this structure was associated with the intention to vote for Donald Trump for U.S. President in 2020. We found that self-centered antagonism and indifference to other people predicted intended vote for Trump over and above salient demographic variables. These findings inform how we might think about the link between personality and voting, and have implications for future research.

Study findings suggest that aggression and indifference are the aspects of narcissism most associated with intending to vote for Donald Trump in 2020 rather than more vulnerable aspects of narcissism. This is consistent with Trump’s aggressive stance during his presidency and (re)election campaigns [27, 29] rather than one that is more consoling towards his supporters and any vulnerability they might feel. It is feasible that vulnerable aspects of narcissism (and/or other aspects of personality) may have predicted voting for Trump in 2016 (e.g., that those who voted for him in 2016 did so out of vulnerability and frustration; see [32]), and that this changed in the 2020 election cycle. However, these findings cannot speak to this directly. Given the chronological variability of pathological aspects of personality vis-à-vis normal personality traits (for review see [59]), an extension of this research could involve a longitudinal investigation of how aspects of pathological narcissism differentially predict likelihood of voting (or a proxy of this, such as level of approval) over time.

Nevertheless, findings may have implications for understanding the role of personality in elections. By all accounts, Donald Trump ran his 2020 reelection campaign and his presidency more broadly based on the dimensions of narcissism highlighted in this study: antagonism and indifference seem to have been guiding principles, both implicitly and explicitly [27–29]. While touching on emotional (and at times darker) aspects of human nature has long been a feature in politicking, especially among the modern-day Republican Party [60], we might reflect on the results of the 2020 presidential election in considering how effective it is to rely on this entirely. That Trump definitively lost both the popular vote (with his opponent Joe Biden winning more votes than any other candidate in American history [61]) as well as the electoral college (which generally favors Republican-leaning states) suggest that doing so may not be that effective: a platform rooted in animosity towards others can generate a substantial amount of angry enthusiasm (as was clear during the election and its immediate aftermath), but may not be one that is convincing to the majority of people, at least not in a country as diverse as the U.S.

In a different vein, findings generally support the results of previous studies on the hierarchical structure of narcissism [14–18]. General narcissism split into grandiose and vulnerable dimensions, and later into dimensions such as antagonism, boldness, and distrust, although some of these factors emerged at different levels of the structural hierarchy than in previous studies. This likely reflects not only a different sample, but also a different selection of narcissism scales than in others studies. However, all scales included in this and previous research on the structure of narcissism included only those measuring *personal* narcissism. This is in contrast to *collective* narcissism, which has been prominent in research on political psychology but not clinical/personality psychology (for review see [32]). Future research on the structure (and, if relevant, predictive validity) of narcissism might thus include a broader measurement of narcissism.

The limitations of this study suggest further avenues of future study. Perhaps foremost among these is the outcome variable: in this study we measured *intended* voting for Donald Trump rather than *actual* voting, which may be problematic in many ways. For example, some study participants who actually intended to vote for Trump may have been reticent to state this in an online survey for fear that the information might become public and they would subsequently be judged by others (a phenomenon similar to the “shy” Trump voter in 2016 who despite their intended and eventual vote for Trump denied this when polled for fear of being judged [62]). It is also possible that participants’ voting intentions changed from the time of data collection to when they cast their votes. Although measuring actual voting is not feasible in the U.S. for ethical reasons, the next-best proxy for this may be exit polls, which tend to correlate highly with election outcomes, although they are also not without problems (for review see [63]). Another useful approach would be to measure intended voting less in terms of categories (e.g., Trump vs. someone else) than in terms like dimensional likelihood of voting for Trump (e.g., very unlikely, somewhat unlikely, somewhat likely, very likely). This approach might have yielded a different and more precise depiction of voters’ voting intensions. In addition, although the sample of this study was representative of the U.S. based on state residence in that residents of most states contributed to the study, it was not as representative of the voting behavior of the nation as a whole. Specifically, participants in this study reported intending to vote for Trump much less (and to a lesser extent, for Biden more) than was reflected in the actual election results. This may be in part due to the tendency of Mturk workers to be better educated than the U.S. population as a whole [64], which in turn was negatively associated with voting for Trump. This study also did not include a measure of socio-economic status, which also has a salient influence on voting behavior (for review see [65]). Future approaches to measuring the influence of narcissism and other aspects of personality on voting studies should thus use a proxy of voting more directly reflecting actual voting and recruiting a sample more representative of the population of interest.

In this study we examined the influence of aspects of pathological narcissism on intention to vote for Donald Trump for U.S. President in 2020, finding that self-centered antagonism and indifference to others were the aspects of narcissism driving intended Trump voting. When considered along with the fact that Trump lost the election, findings suggest that appealing to the darker aspects of personality may not be the most effective way to win elections.

## Supporting information

S1 File (DOCX)
