# Supplementary material for: Dimensions of pathological narcissism and intention to vote for Donald Trump
Source: PLoS One. 2021 Apr 15;16(4):e0249892. doi: 10.1371/journal.pone.0249892 (PMC8049239; doi:10.1371/journal.pone.0249892)
Supplement: S1 File — (DOCX) [file pone.0249892.s001.docx]

We conducted a series of exploratory factor analyses (EFAs) to confirm the structure of individual measures of pathological narcissism (the FFNI, PNI, NARQ, and HSNS). We conducted each EFA using maximum likelihood extraction and oblique promax rotation in order to allow factors to correlate with each other, consistent with measures’ designs. In each case, the EFA yielded a factor solution that resembled the measures’ structures demonstrated in previous studies (see S11-S14 Tables).

**List of Tables**

- S1 Table. *Participants Representation by State*
- S2 Table. *Correlations Between and Internal Consistencies for Study Variables*
- S3 Table. *Intended Vote for Trump by Sex*
- S4 Table. *Intended Vote for Trump by Latinx Identification*
- S5 Table. *Intended Vote for Trump by Sexual Orientation*
- S6 Table. *Intended Vote for Trump by Biological Sex*
- S7 Table. *Intended Vote for Trump by Gender*
- S8 Table. *Intended Vote for Trump by Education Level*
- S9 Table. *Eigenvalues, Variance Accounted for, and Loadings for Hierarchical PCA*
- S10 Table. *Incremental Effects of Dimensions Pathological Narcissism on Voting for Donald Trump in 2020*
- S11 Table. *Eigenvalues, Variance Accounted for, and Loadings for EFA for the FFNI*
- S12 Table. *Eigenvalues, Variance Accounted for, and Loadings for EFA for the PNI*
- S13 Table. *Eigenvalues, Variance Accounted for, and Loadings for EFA for the NARQ*
- S14 Table. *Eigenvalues, Variance Accounted for, and Loadings for EFA for the HSNS*

**S1 Table**

*Participants Representation by State*

| State | *n* (%) | State | *n* (%) |
| --- | --- | --- | --- |
| Alabama | 12 (2%) | Missouri | 8 (2%) |
| Arizona | 11 (2%) | Montana | 3 (1%) |
| Arkansas | 5 (1%) | Nevada | 5 (1%) |
| California | 57 (12%) | New Jersey | 16 (3%) |
| Colorado | 8 (2%) | New Mexico | 1 (< 1%) |
| Connecticut | 11 (2%) | New York | 23 (5%) |
| Delaware | 3 (1%) | North Carolina | 25 (5%) |
| Florida | 37 (7%) | Ohio | 16 (3%) |
| Georgia | 13 (3%) | Oklahoma | 2 (< 1%) |
| Hawaii | 1 (< 1%) | Oregon | 7 (1%) |
| Idaho | 4 (1%) | Pennsylvania | 19 (4%) |
| Illinois | 22 (4%) | Rhode Island | 2 (< 1%) |
| Indiana | 10 (2%) | South Carolina | 9 (2%) |
| Iowa | 3 (1%) | South Dakota | 2 (< 1%) |
| Kansas | 7 (1%) | Tennessee | 7 (1%) |
| Kentucky | 12 (2%) | Texas | 37 (7%) |
| Louisiana | 5 (1%) | Utah | 2 (< 1%) |
| Maine | 5 (1%) | Virginia | 13 (3%) |
| Maryland | 14 (3%) | Washington | 8 (2%) |
| Massachusetts | 8 (2%) | West Virginia | 2 (< 1%) |
| Michigan | 14 (3%) | Wisconsin | 5 (1%) |
| Minnesota | 9 (2%) | Wyoming | 1 (< 1%) |
| Mississippi | 6 (1%) | District of Columbia | 1 (< 1%) |

**S2 Table**

*Correlations Between and Internal Consistencies for Study Variables*

|  | 1 | 2 | 3 | 4 | 5 | 6 | 7 | 8 |
| --- | --- | --- | --- | --- | --- | --- | --- | --- |
| 1. Voting for Trump | — |  |  |  |  |  |  |  |
| 2. Age | .02 | — |  |  |  |  |  |  |
| 3. Male sex | .01 | -.11 | — |  |  |  |  |  |
| 4. White race | .01 | .12 | -.11 | — |  |  |  |  |
| 5. Heterosexual orientation | -.04 | .08 | .05 | .12 | — |  |  |  |
| 6. College degree | .04 | -.09 | .06 | -.13 | -.02 | — |  |  |
| 7. Red state | .02 | .06 | -.05 | .09 | .01 | -.13 | — |  |
| 8. Republican Party | .70 | .06 | .01 | -.05 | -.05 | .09 | .02 | — |
| 9. FFNI Acclaim-Seeking | .15 | -.30 | .13 | -.12 | -.03 | .13 | -.10 | .17 |
| 10. FFNI Arrogance | .28 | -.21 | .18 | -.11 | -.18 | .25 | -.13 | .25 |
| 11. FFNI Authortativeness | .16 | -.14 | .15 | -.07 | -.12 | .22 | -.03 | .16 |
| 12. FFNI Distrust | .12 | -.16 | .07 | -.08 | -.12 | .01 | -.04 | .11 |
| 13. FFNI Entitlement | .25 | -.24 | .15 | -.21 | -.17 | .28 | -.11 | .23 |
| 14. FFNI Exhibitionism | .20 | -.18 | .15 | -.06 | -.19 | .20 | -.07 | .17 |
| 15. FFNI Exploitativeness | .25 | -.24 | .21 | -.12 | -.23 | .26 | -.09 | .20 |
| 16. FFNI Grandiose Fantasies | .15 | -.36 | .18 | -.15 | -.17 | .20 | -.09 | .13 |
| 17. FFNI Indifference | .18 | -.13 | .22 | -.13 | -.14 | .12 | -.08 | .14 |
| 18. FFNI Lack of Empathy | .29 | -.18 | .23 | -.14 | -.17 | .18 | -.07 | .25 |
| 19. FFNI Manipulativeness | .20 | -.23 | .18 | -.06 | -.25 | .16 | -.04 | .16 |
| 20. FFNI Need for Admiration | .11 | -.24 | .01 | -.02 | -.17 | .14 | -.04 | .11 |
| 21. FFNI Reactive Anger | .23 | -.25 | .12 | -.06 | -.21 | .18 | -.10 | .21 |
| 22. FFNI Shame | .09 | -.12 | -.08 | .02 | -.16 | .06 | .03 | .06 |
| 23. FFNI Thrill-Seeking | .25 | -.30 | .21 | -.13 | -.27 | .23 | -.07 | .19 |
| 24. NARQ Grandiosity | .21 | -.29 | .13 | -.15 | -.15 | .24 | -.10 | .19 |
| 25. NARQ Uniqueness | .18 | -.22 | .14 | -.11 | -.16 | .21 | -.10 | .17 |
| 26. NARQ Charmingness | .21 | -.15 | .16 | -.08 | -.17 | .22 | -.12 | .19 |
| 27. NARQ Devaluation | .26 | -.19 | .23 | -.09 | -.18 | .23 | -.09 | .21 |
| 28. NARQ Supremacy | .25 | -.21 | .19 | -.11 | -.17 | .18 | -.08 | .19 |
| 29. NARQ Aggressiveness | .26 | -.19 | .13 | -.04 | -.21 | .25 | -.09 | .22 |
| 30. PNI Contingent Self-Esteem | .21 | -.25 | .09 | -.07 | -.21 | .22 | -.05 | .19 |
| 31. PNI Exploitative | .19 | -.22 | .16 | -.07 | -.23 | .14 | -.07 | .16 |
| 32. PNI Self-Sacrificing Self-Enhancement | .15 | -.20 | .09 | -.01 | -.14 | .19 | -.07 | .16 |
| 33. PNI Hiding the Self | .16 | -.07 | .05 | .04 | -.17 | .03 | -.03 | .12 |
| 34. PNI Grandiose Fantasy | .15 | -.32 | .15 | -.10 | -.19 | .20 | -.10 | .14 |
| 35. PNI Devaluing | .23 | -.25 | .14 | -.06 | -.23 | .20 | -.06 | .19 |
| 36. PNI Entitlement Rage | .27 | -.24 | .14 | -.07 | -.19 | .21 | -.10 | .24 |
| 37. HSNS | -.06 | -.05 | .04 | .01 | .05 | -.09 | .00 | -.06 |

*Note:* Unless otherwise noted, *r*s ≥ |.09| are statistically significant at *p* < .05; † indicates *r* is not statistically significant at *p* < 0.5.

**S2 Table** (continued)

*Correlations Between and Internal Consistencies for Study Variables*

|  | 9 | 10 | 11 | 12 | 13 | 14 | 15 | 16 |
| --- | --- | --- | --- | --- | --- | --- | --- | --- |
| 9. FFNI Acclaim-Seeking | — |  |  |  |  |  |  |  |
| 10. FFNI Arrogance | .49 | — |  |  |  |  |  |  |
| 11. FFNI Authortativeness | .64 | .51 | — |  |  |  |  |  |
| 12. FFNI Distrust | .10 | .35 | .06 | — |  |  |  |  |
| 13. FFNI Entitlement | .44 | .83 | .43 | .32 | — |  |  |  |
| 14. FFNI Exhibitionism | .51 | .58 | .57 | .07 | .60 | — |  |  |
| 15. FFNI Exploitativeness | .38 | .80 | .43 | .37 | .82 | .57 | — |  |
| 16. FFNI Grandiose Fantasies | .50 | .67 | .44 | .29 | .71 | .62 | .67 | — |
| 17. FFNI Indifference | .34 | .47 | .45 | .26 | .43 | .32 | .44 | .32 |
| 18. FFNI Lack of Empathy | .26 | .72 | .32 | .48 | .69 | .31 | .75 | .49 |
| 19. FFNI Manipulativeness | .43 | .71 | .62 | .31 | .66 | .64 | .74 | .63 |
| 20. FFNI Need for Admiration | .09^‡^ | .44 | -.02 | .35 | .46 | .29 | .43 | .38 |
| 21. FFNI Reactive Anger | .33 | .72 | .36 | .45 | .70 | .50 | .69 | .59 |
| 22. FFNI Shame | -.02 | .22 | -.10 | .29 | .24 | .14 | .24 | .19 |
| 23. FFNI Thrill-Seeking | .45 | .74 | .44 | .36 | .75 | .59 | .77 | .64 |
| 24. NARQ Grandiosity | .59 | .75 | .58 | .19 | .76 | .65 | .66 | .71 |
| 25. NARQ Uniqueness | .58 | .69 | .57 | .19 | .66 | .64 | .61 | .62 |
| 26. NARQ Charmingness | .53 | .67 | .65 | .15 | .67 | .72 | .64 | .59 |
| 27. NARQ Devaluation | .24 | .73 | .30 | .44 | .72 | .44 | .78 | .58 |
| 28. NARQ Supremacy | .32 | .73 | .35 | .47 | .69 | .46 | .77 | .59 |
| 29. NARQ Aggressiveness | .32 | .73 | .35 | .39 | .74 | .55 | .75 | .61 |
| 30. PNI Contingent Self-Esteem | .31 | .68 | .24 | .36 | .69 | .54 | .68 | .59 |
| 31. PNI Exploitative | .48 | .67 | .64 | .24 | .64 | .67 | .67 | .62 |
| 32. PNI Self-Sacrificing Self-Enhancement | .48 | .48 | .44 | .07 | .45 | .59 | .40 | .47 |
| 33. PNI Hiding the Self | .13 | .35 | .08 | .48 | .32 | .17 | .36 | .26 |
| 34. PNI Grandiose Fantasy | .52 | .62 | .43 | .32 | .62 | .63 | .58 | .80 |
| 35. PNI Devaluing | .31 | .69 | .27 | .49 | .69 | .47 | .69 | .60 |
| 36. PNI Entitlement Rage | .38 | .76 | .38 | .48 | .75 | .55 | .75 | .62 |
| 37. HSNS | -.19 | .04 | -.17 | .53 | .03 | -.21 | .12 | .05 |

*Note:* Unless otherwise noted, *r*s ≥ |.09| are statistically significant at *p* < .05; ^‡^ indicates *r* is not statistically significant at *p* < 0.5.

**S2 Table** (continued)

*Correlations Between and Internal Consistencies for Study Variables*

|  | 17 | 18 | 19 | 20 | 21 | 22 | 23 | 24 | 25 | 26 | 27 | 28 |
| --- | --- | --- | --- | --- | --- | --- | --- | --- | --- | --- | --- | --- |
| 17. FFNI Indifference | — |  |  |  |  |  |  |  |  |  |  |  |
| 18. FFNI Lack of Empathy | .53 | — |  |  |  |  |  |  |  |  |  |  |
| 19. FFNI Manipulativeness | .52 | .60 | — |  |  |  |  |  |  |  |  |  |
| 20. FFNI Need for Admiration | -.12 | .34 | .30 | — |  |  |  |  |  |  |  |  |
| 21. FFNI Reactive Anger | .29 | .59 | .63 | .58 | — |  |  |  |  |  |  |  |
| 22. FFNI Shame | -.23 | .17 | .16 | .67 | .48 | — |  |  |  |  |  |  |
| 23. FFNI Thrill-Seeking | .47 | .64 | .70 | .44 | .66 | .20 | — |  |  |  |  |  |
| 24. NARQ Grandiosity | .47 | .53 | .66 | .25 | .57 | .07 | .65 | — |  |  |  |  |
| 25. NARQ Uniqueness | .42 | .45 | .62 | .22 | .53 | .11 | .57 | .81 | — |  |  |  |
| 26. NARQ Charmingness | .46 | .46 | .71 | .23 | .54 | .11 | .61 | .73 | .75 | — |  |  |
| 27. NARQ Devaluation | .36 | .73 | .59 | .49 | .66 | .33 | .69 | .55 | .51 | .54 | — |  |
| 28. NARQ Supremacy | .35 | .70 | .64 | .45 | .68 | .33 | .62 | .59 | .61 | .59 | .76 | — |
| 29. NARQ Aggressiveness | .25 | .63 | .61 | .59 | .80 | .50 | .68 | .61 | .59 | .62 | .74 | .76 |
| 30. PNI Contingent Self-Esteem | .10 | .52 | .54 | .79 | .76 | .60 | .68 | .54 | .50 | .53 | .70 | .65 |
| 31. PNI Exploitative | .50 | .49 | .88 | .25 | .56 | .10 | .66 | .71 | .68 | .79 | .53 | .60 |
| 32. PNI Self-Sacrificing Self-Enhancement | .20 | .21 | .45 | .39 | .46 | .34 | .46 | .52 | .61 | .60 | .37 | .39 |
| 33. PNI Hiding the Self | .14 | .38 | .33 | .52 | .50 | .62 | .36 | .19 | .25 | .25 | .45 | .47 |
| 34. PNI Grandiose Fantasy | .26 | .42 | .60 | .50 | .61 | .36 | .63 | .65 | .66 | .61 | .56 | .57 |
| 35. PNI Devaluing | .22 | .56 | .56 | .67 | .77 | .55 | .69 | .55 | .52 | .53 | .71 | .68 |
| 36. PNI Entitlement Rage | .26 | .64 | .66 | .60 | .86 | .49 | .68 | .62 | .60 | .63 | .73 | .75 |
| 37. HSNS | .03 | .32 | .06 | .08 | .12 | .03 | .01 | -.12 | -.15 | -.13 | .21 | .26 |

*Note:* Unless otherwise noted, *r*s ≥ |.09| are statistically significant at *p* < .05; ^‡^ indicates *r* is not statistically significant at *p* < 0.5.

**S2 Table** (continued)

*Correlations Between and Internal Consistencies for Study Variables*

|  | 29 | 30 | 31 | 32 | 33 | 34 | 35 | 36 | 37 |
| --- | --- | --- | --- | --- | --- | --- | --- | --- | --- |
| 29. NARQ Aggressiveness | — |  |  |  |  |  |  |  |  |
| 30. PNI Contingent Self-Esteem | .83 | — |  |  |  |  |  |  |  |
| 31. PNI Exploitative | .58 | .52 | — |  |  |  |  |  |  |
| 32. PNI Self-Sacrificing Self-Enhancement | .51 | .60 | .55 | — |  |  |  |  |  |
| 33. PNI Hiding the Self | .50 | .58 | .30 | .42 | — |  |  |  |  |
| 34. PNI Grandiose Fantasy | .65 | .70 | .63 | .65 | .47 | — |  |  |  |
| 35. PNI Devaluing | .81 | .87 | .54 | .56 | .64 | .68 | — |  |  |
| 36. PNI Entitlement Rage | .86 | .85 | .65 | .56 | .58 | .69 | .85 | — |  |
| 37. HSNS | .09 | -.02 | -.04 | -.36 | .16 | -.01 | .10 | .12 | — |

*Note:* Unless otherwise noted, *r*s ≥ |.09| are statistically significant at *p* < .05; ^‡^ indicates *r* is not statistically significant at *p* < 0.5.

**S3 Table**

*Intended Vote for Trump by Sex*

|  |  | Political Party | | | | | |
| --- | --- | --- | --- | --- | --- | --- | --- |
|  |  | Democratic | Republican | Libertarian | Socialist | Independent | Other |
| Intended vote for Trump | No | 190^a^ | 20^b^ | 2^b,c^ | 7^a,c^ | 65^a,c^ | 10^a,c^ |
|  | Yes | 27^a^ | 150^b^ | 3^b,c^ | 0^a,c^ | 21^a,c^ | 0^a,c^ |

*Note.* Different superscripts indicate statistically significant comparisons between groups with Bonferroni *post hoc* correction (e.g., values with the superscript “a” are significantly different from values with the superscript “b” and values with the superscript “a, b” are not significantly different from values with superscripts “a” or “b”).

**S4 Table**

*Intended Vote for Trump by Latinx Identification*

|  |  | Latinx | |
| --- | --- | --- | --- |
|  |  | Yes | No |
| Intended vote for Trump | No | 21^a^ | 273^b^ |
|  | Yes | 26^a^ | 175^b^ |

*Note.* Different superscripts indicate statistically significant comparisons between groups with Bonferroni *post hoc* correction (e.g., values with the superscript “a” are significantly different from values with the superscript “b” and values with the superscript “a, b” are not significantly different from values with superscripts “a” or “b”).

**S5 Table**

*Intended Vote for Trump by Sexual Orientation*

|  |  | Sexual Orientation | | | |
| --- | --- | --- | --- | --- | --- |
|  |  | Heterosexual | Gay/Lesbian | Bisexual | Other |
| Intended vote for Trump | No | 253^a,b^ | 13^b^ | 23^a^ | 5^a,b^ |
|  | Yes | 167^a,b^ | 2^b^ | 32^a^ | 0^a,b^ |

*Note.* Different superscripts indicate statistically significant comparisons between groups with Bonferroni *post hoc* correction (e.g., values with the superscript “a” are significantly different from values with the superscript “b” and values with the superscript “a, b” are not significantly different from values with superscripts “a” or “b”).

**S6 Table**

*Intended Vote for Trump by Biological Sex*

|  |  | Sex | | | |
| --- | --- | --- | --- | --- | --- |
|  |  | Male | Female | Intersex | Other (not listed) |
| Intended vote for Trump | No | 155 | 136 | 1 | 1 |
|  | Yes | 108 | 92 | 0 | 0 |

**S7 Table**

*Intended Vote for Trump by Gender*

|  |  | Gender | | | | |
| --- | --- | --- | --- | --- | --- | --- |
|  |  | Cisgender Man | Cisgender Woman | Trans Woman | Genderqueer | Other (not listed) |
| Intended vote for Trump | No | 154 | 137 | 1 | 1 | 1 |
|  | Yes | 110 | 91 | 0 | 0 | 0 |

**S8 Table**

*Intended Vote for Trump by Education Level*

|  |  | Education Level | | | | | |
| --- | --- | --- | --- | --- | --- | --- | --- |
|  |  | Some schooling | High school/GED | Some college | Bachelor's degree | Master's degree | Doctorate |
| Intended vote for Trump | No | 2 | 31 | 61 | 147 | 46 | 7 |
|  | Yes | 0 | 18 | 39 | 105 | 37 | 1 |

**S9 Table**

*Eigenvalues, Variance Accounted for, and Loadings for Hierarchical PCA*

|  | 1 | 2 | | 3 | | |
| --- | --- | --- | --- | --- | --- | --- |
|  | NAR | GRN | VLN | GRN | VLN | DIS |
| Eigenvalue | 15.45 | 15.45 | 3.38 | 15.45 | 3.38 | 2.14 |
| % of variance | 53.26 | 53.26 | 11.65 | 53.26 | 11.65 | 7.37 |
| FFNI Acclaim-Seeking | **.54** | **.71** | -.04 | **.72** | .04 | -.11 |
| FFNI Arrogance | **.88** | **.74** | **.48** | **.72** | **.37** | **.38** |
| FFNI Authortativeness | **.55** | **.79** | -.11 | **.80** | -.09 | -.01 |
| FFNI Distrust | **.44** | .07 | **.62** | .03 | **.35** | **.67** |
| FFNI Entitlement | **.87** | **.71** | **.50** | **.69** | **.40** | **.36** |
| FFNI Exhibitionism | **.71** | **.78** | .16 | **.78** | .26 | -.10 |
| FFNI Exploitativeness | **.87** | **.68** | **.54** | **.65** | **.38** | **.48** |
| FFNI Grandiose Fantasies | **.78** | **.69** | **.38** | **.68** | **.35** | .20 |
| FFNI Indifference | **.46** | **.61** | -.04 | **.59** | -.25 | **.44** |
| FFNI Lack of Empathy | **.72** | **.49** | **.54** | **.45** | .25 | **.71** |
| FFNI Manipulativeness | **.81** | **.77** | **.33** | **.75** | .23 | .33 |
| FFNI Need for Admiration | **.56** | **.08** | **.80** | .07 | **.86** | .10 |
| FFNI Reactive Anger | **.83** | **.50** | **.70** | **.48** | **.63** | **.35** |
| FFNI Shame | **.38** | -.11 | **.75** | -.11 | **.86** | -.02 |
| FFNI Thrill-Seeking | **.83** | **.68** | **.47** | **.66** | **.38** | **.34** |
| NARQ Grandiosity | **.80** | **.85** | .21 | **.84** | .20 | .14 |
| NARQ Uniqueness | **.77** | **.82** | .20 | **.82** | .22 | .06 |
| NARQ Charmingness | **.78** | **.85** | .19 | **.84** | .21 | .07 |
| NARQ Devaluation | **.80** | **.50** | **.66** | **.46** | **.49** | **.53** |
| NARQ Supremacy | **.82** | **.54** | **.63** | **.51** | **.46** | **.52** |
| NARQ Aggressiveness | **.87** | **.54** | **.72** | **.51** | **.67** | **.31** |
| PNI Contingent Self-Esteem | **.83** | **.44** | **.79** | **.42** | **.83** | .14 |
| PNI Exploitative | **.79** | **.83** | .23 | **.82** | .20 | .19 |
| PNI Self-Sacrificing Self-Enhancement | **.64** | **.59** | .28 | **.61** | **.50** | **-.31** |
| PNI Hiding the Self | **.52** | .09 | **.72** | .07 | **.70** | .24 |
| PNI Grandiose Fantasy | **.80** | **.64** | **.48** | **.63** | **.54** | .05 |
| PNI Devaluing | **.84** | **.44** | **.80** | **.42** | **.76** | .29 |
| PNI Entitlement Rage | **.89** | **.56** | **.74** | **.53** | **.68** | **.33** |
| HSNS | .05 | -.24 | **.38** | -.28 | .02 | **.76** |

*Note:* Loadings ≥ .30 in **boldface** font; NAR = overall narcissism, GRN = grandiosity; VLN = vulnerability, DIS = distrust.

**S9 Table** (continued)

*Eigenvalues, Variance Accounted for, and Loadings for Hierarchical PCA*

|  | 4 | | | |
| --- | --- | --- | --- | --- |
|  | ANT | GRN | VLN | DIS |
| Eigenvalue | 15.45 | 3.38 | 2.14 | 1.00 |
| % of variance | 53.26 | 11.65 | 7.37 | 3.45 |
| FFNI Acclaim-Seeking | .10 | **.77** | .06 | -.01 |
| FFNI Arrogance | **.74** | **.47** | .21 | .10 |
| FFNI Authortativeness | .18 | **.82** | -.09 | .04 |
| FFNI Distrust | .22 | .07 | **.33** | **.77** |
| FFNI Entitlement | **.79** | **.40** | .22 | .02 |
| FFNI Exhibitionism | **.40** | **.66** | .20 | -.23 |
| FFNI Exploitativeness | **.82** | **.35** | .20 | .14 |
| FFNI Grandiose Fantasies | **.55** | **.52** | .24 | .03 |
| FFNI Indifference | **.33** | **.53** | **-.31** | **.38** |
| FFNI Lack of Empathy | **.75** | .19 | .08 | **.42** |
| FFNI Manipulativeness | **.55** | **.62** | .12 | .19 |
| FFNI Need for Admiration | **.39** | -.06 | **.78** | -.02 |
| FFNI Reactive Anger | **.60** | **.30** | **.51** | .17 |
| FFNI Shame | .10 | -.10 | **.86** | .03 |
| FFNI Thrill-Seeking | **.68** | **.44** | .24 | .10 |
| NARQ Grandiosity | **.58** | **.66** | .08 | -.06 |
| NARQ Uniqueness | **.44** | **.72** | .15 | -.04 |
| NARQ Charmingness | **.47** | **.72** | .13 | -.04 |
| NARQ Devaluation | **.76** | .19 | **.32** | .22 |
| NARQ Supremacy | **.68** | **.30** | **.31** | **.30** |
| NARQ Aggressiveness | **.68** | .29 | **.52** | .07 |
| PNI Contingent Self-Esteem | **.58** | .24 | **.72** | -.04 |
| PNI Exploitative | **.46** | **.72** | .13 | .10 |
| PNI Self-Sacrificing Self-Enhancement | .10 | **.65** | **.53** | -.21 |
| PNI Hiding the Self | .07 | .18 | **.73** | **.43** |
| PNI Grandiose Fantasy | **.35** | **.59** | **.51** | .04 |
| PNI Devaluing | **.54** | .28 | **.66** | .16 |
| PNI Entitlement Rage | **.61** | **.37** | **.57** | .18 |
| HSNS | .18 | **-.30** | -.03 | **.76** |

*Note:* Loadings ≥ .30 in **boldface** font; ANT = antagonism, GRN = grandiosity; VLN = vulnerability, DIS = distrust.

**S9 Table** (continued)

*Eigenvalues, Variance Accounted for, and Loadings for Hierarchical PCA*

|  | 5 | | | | |
| --- | --- | --- | --- | --- | --- |
|  | ANT | GRN | VLN | DIS | IND |
| Eigenvalue | 15.45 | 3.38 | 2.14 | 1.00 | .82 |
| % of variance | 53.26 | 11.65 | 7.37 | 3.45 | 2.82 |
| FFNI Acclaim-Seeking | .08 | **.82** | .02 | .04 | .03 |
| FFNI Arrogance | **.75** | **.45** | .21 | .05 | .10 |
| FFNI Authortativeness | .19 | **.75** | -.08 | -.06 | **.32** |
| FFNI Distrust | .23 | .07 | **.36** | **.75** | .18 |
| FFNI Entitlement | **.79** | **.42** | .20 | .02 | -.02 |
| FFNI Exhibitionism | **.39** | **.69** | .15 | -.21 | -.03 |
| FFNI Exploitativeness | **.83** | **.33** | .20 | .09 | .09 |
| FFNI Grandiose Fantasies | **.50** | **.65** | .16 | .19 | -.26 |
| FFNI Indifference | **.40** | **.34** | -.21 | .12 | **.66** |
| FFNI Lack of Empathy | **.78** | .13 | .12 | **.31** | .26 |
| FFNI Manipulativeness | **.58** | **.54** | .15 | .07 | **.31** |
| FFNI Need for Admiration | **.38** | .03 | **.74** | .04 | -.28 |
| FFNI Reactive Anger | **.61** | **.31** | **.51** | .13 | .04 |
| FFNI Shame | .11 | -.07 | **.86** | .00 | -.11 |
| FFNI Thrill-Seeking | **.69** | **.42** | .24 | .05 | .10 |
| NARQ Grandiosity | **.57** | **.69** | .04 | -.04 | .01 |
| NARQ Uniqueness | **.43** | **.72** | .13 | -.06 | .08 |
| NARQ Charmingness | **.48** | **.67** | .14 | -.13 | .22 |
| NARQ Devaluation | **.78** | .18 | **.32** | .17 | .07 |
| NARQ Supremacy | **.69** | **.30** | **.32** | .26 | .10 |
| NARQ Aggressiveness | **.68** | **.31** | **.51** | .05 | -.02 |
| PNI Contingent Self-Esteem | **.57** | .28 | **.69** | -.04 | -.14 |
| PNI Exploitative | **.48** | **.65** | .14 | -.01 | **.30** |
| PNI Self-Sacrificing Self-Enhancement | .12 | **.62** | **.52** | -.29 | .11 |
| PNI Hiding the Self | .12 | .09 | **.80** | .25 | **.33** |
| PNI Grandiose Fantasy | **.32** | **.69** | **.44** | .14 | -.15 |
| PNI Devaluing | **.55** | **.30** | **.66** | .13 | .00 |
| PNI Entitlement Rage | **.62** | **.37** | **.56** | .13 | .05 |
| HSNS | .16 | -.22 | -.04 | **.86** | -.06 |

*Note:* Loadings ≥ . 30 in **boldface** font; ANT = antagonism, GRN = grandiosity; VLN = vulnerability, DIS = distrust, IND = indifference.

**S9 Table** (continued)

*Eigenvalues, Variance Accounted for, and Loadings for Hierarchical PCA*

|  | 6 | | | | | |
| --- | --- | --- | --- | --- | --- | --- |
|  | ANT | BLD | VLN | GRN | DIS | IND |
| Eigenvalue | 15.45 | 3.38 | 2.14 | 1.00 | .82 | .68 |
| % of variance | 53.26 | 11.65 | 7.37 | 3.45 | 2.82 | 2.36 |
| FFNI Acclaim-Seeking | .16 | **.31** | .01 | **.80** | -.07 | .17 |
| FFNI Arrogance | **.76** | **.34** | .21 | **.30** | .02 | .13 |
| FFNI Authortativeness | .15 | **.69** | -.08 | **.39** | -.06 | .24 |
| FFNI Distrust | .25 | -.01 | **.37** | .14 | **.71** | .25 |
| FFNI Entitlement | **.80** | .29 | .20 | .29 | -.02 | .01 |
| FFNI Exhibitionism | **.34** | **.67** | .15 | **.33** | -.18 | -.16 |
| FFNI Exploitativeness | **.80** | **.38** | .20 | .12 | .09 | .06 |
| FFNI Grandiose Fantasies | **.52** | **.37** | .16 | **.54** | .16 | -.23 |
| FFNI Indifference | **.40** | **.34** | -.20 | .16 | .08 | **.69** |
| FFNI Lack of Empathy | **.79** | .15 | .13 | .06 | .28 | **.31** |
| FFNI Manipulativeness | **.48** | **.76** | .16 | .09 | .13 | .12 |
| FFNI Need for Admiration | **.38** | -.02 | **.74** | .06 | .04 | -.27 |
| FFNI Reactive Anger | **.59** | **.30** | **.51** | .16 | .12 | .01 |
| FFNI Shame | .10 | -.03 | **.86** | -.07 | .01 | -.13 |
| FFNI Thrill-Seeking | **.69** | **.34** | .24 | .26 | .02 | .12 |
| NARQ Grandiosity | **.58** | **.45** | .04 | **.51** | -.09 | .04 |
| NARQ Uniqueness | **.44** | **.51** | .13 | **.51** | -.10 | .09 |
| NARQ Charmingness | **.42** | **.72** | .14 | .27 | -.11 | .09 |
| NARQ Devaluation | **.77** | .21 | **.33** | .06 | .16 | .07 |
| NARQ Supremacy | **.66** | **.35** | **.32** | .12 | .27 | .06 |
| NARQ Aggressiveness | **.66** | **.32** | **.52** | .13 | .05 | -.06 |
| PNI Contingent Self-Esteem | **.57** | .21 | **.69** | .19 | -.05 | -.14 |
| PNI Exploitative | **.39** | **.82** | .15 | .17 | .04 | .11 |
| PNI Self-Sacrificing Self-Enhancement | .12 | **.44** | **.52** | **.43** | **-.34** | .10 |
| PNI Hiding the Self | .11 | .10 | **.80** | .05 | .22 | **.34** |
| PNI Grandiose Fantasy | **.33** | **.41** | **.44** | **.56** | .10 | -.13 |
| PNI Devaluing | **.56** | .21 | **.66** | .22 | .10 | .03 |
| PNI Entitlement Rage | **.60** | **.36** | **.57** | .19 | .13 | .02 |
| HSNS | .13 | -.09 | -.02 | -.16 | **.91** | -.08 |

*Note:* Loadings ≥ . 30 in **boldface** font; ANT = antagonism, BLD = boldness, GRN = grandiosity; DIS = distrust, VLN = vulnerability, IND = indifference.

.

**S9 Table** (continued)

*Eigenvalues, Variance Accounted for, and Loadings for Hierarchical PCA*

|  |  |  |  |  |  |  |  |
| --- | --- | --- | --- | --- | --- | --- | --- |
|  | 7 | | | | | | |
|  | ANT | VLN | BLD | AS | DIS | GRN | IND |
| Eigenvalue | 15.45 | 3.38 | 2.14 | 1.00 | .82 | .68 | .57 |
| % of variance | 53.26 | 11.65 | 7.37 | 3.45 | 2.82 | 2.36 | 1.96 |
| FFNI Acclaim-Seeking | .16 | .02 | .23 | **.84** | -.05 | .15 | .12 |
| FFNI Arrogance | **.76** | .20 | .30 | **.32** | .02 | .12 | .14 |
| FFNI Authortativeness | .19 | -.07 | **.64** | **.54** | -.04 | -.09 | .14 |
| FFNI Distrust | .23 | **.38** | -.01 | .10 | **.71** | .09 | .28 |
| FFNI Entitlement | **.78** | .19 | .26 | .25 | -.03 | .25 | .09 |
| FFNI Exhibitionism | **.31** | .15 | **.64** | .27 | -.20 | **.34** | -.03 |
| FFNI Exploitativeness | **.79** | .20 | **.36** | .11 | .08 | .16 | .12 |
| FFNI Grandiose Fantasies | **.46** | .15 | **.35** | **.37** | .13 | **.59** | -.02 |
| FFNI Indifference | **.35** | -.18 | **.31** | .18 | .06 | .00 | **.77** |
| FFNI Lack of Empathy | **.78** | .13 | .13 | .08 | .28 | -.01 | **.31** |
| FFNI Manipulativeness | **.46** | .16 | **.75** | .09 | .11 | .15 | .21 |
| FFNI Need for Admiration | **.36** | **.73** | -.01 | -.08 | .01 | .31 | -.16 |
| FFNI Reactive Anger | **.61** | **.50** | .28 | .17 | .13 | .08 | .00 |
| FFNI Shame | .13 | **.85** | -.02 | -.07 | .01 | -.03 | -.19 |
| FFNI Thrill-Seeking | **.63** | .24 | **.32** | .15 | -.01 | **.37** | **.30** |
| NARQ Grandiosity | **.58** | .04 | **.40** | **.51** | -.09 | .22 | .06 |
| NARQ Uniqueness | **.46** | .12 | **.45** | **.58** | -.08 | .06 | .03 |
| NARQ Charmingness | **.44** | .13 | **.68** | **.35** | -.11 | .04 | .06 |
| NARQ Devaluation | **.77** | .32 | .20 | .05 | .16 | .10 | .11 |
| NARQ Supremacy | **.70** | **.31** | **.32** | .19 | .28 | -.04 | -.01 |
| NARQ Aggressiveness | **.69** | **.50** | **.30** | .16 | .05 | .06 | -.09 |
| PNI Contingent Self-Esteem | **.57** | **.68** | .20 | .11 | -.07 | .26 | -.07 |
| PNI Exploitative | **.37** | .15 | **.80** | .19 | .03 | .15 | .18 |
| PNI Self-Sacrificing Self-Enhancement | .12 | **.53** | **.40** | **.44** | **-.34** | .14 | .10 |
| PNI Hiding the Self | .12 | **.82** | .09 | .08 | .22 | -.08 | .29 |
| PNI Grandiose Fantasy | .29 | **.44** | **.38** | **.42** | .08 | **.51** | .02 |
| PNI Devaluing | **.55** | **.66** | .19 | .16 | .09 | .20 | .07 |
| PNI Entitlement Rage | **.62** | **.56** | **.34** | .22 | .13 | .07 | -.01 |
| HSNS | .14 | -.03 | -.06 | -.18 | **.91** | .00 | -.08 |

*Note:* Loadings ≥ . 30 in **boldface** font; ANT = antagonism, VLN = vulnerability, BLD = boldnessAS = attention-seeking, DIS = distrust, , GRN = grandiosity; VLN = vulnerability, IND = indifference.

**S9 Table** (continued)

*Eigenvalues, Variance Accounted for, and Loadings for Hierarchical PCA*

|  | 8 | | | | | | | |
| --- | --- | --- | --- | --- | --- | --- | --- | --- |
|  | ANT | VLN | BLD | GRN | AS | DIS | IND | UNQ |
| Eigenvalue | 15.45 | 3.38 | 2.14 | 1.00 | .82 | .68 | .57 | .54 |
| % of variance | 53.26 | 11.65 | 7.37 | 3.45 | 2.82 | 2.36 | 1.96 | 1.86 |
| FFNI Acclaim-Seeking | .19 | .03 | .25 | .27 | **.85** | -.08 | .09 | .04 |
| FFNI Arrogance | **.77** | .21 | **.30** | .18 | .27 | .01 | .13 | .07 |
| FFNI Authortativeness | .20 | -.06 | **.66** | -.03 | **.60** | -.06 | .10 | .04 |
| FFNI Distrust | .23 | **.38** | -.01 | .10 | .12 | **.70** | .28 | -.02 |
| FFNI Entitlement | **.79** | .19 | .26 | **.30** | .17 | -.03 | .08 | .01 |
| FFNI Exhibitionism | **.32** | .14 | **.63** | **.40** | .18 | -.21 | -.04 | .00 |
| FFNI Exploitativeness | **.79** | .20 | **.36** | .17 | .07 | .08 | .11 | -.01 |
| FFNI Grandiose Fantasies | **.47** | .14 | **.32** | **.68** | .20 | .11 | .00 | .02 |
| FFNI Indifference | **.37** | -.19 | **.31** | .04 | .15 | .05 | **.77** | .04 |
| FFNI Lack of Empathy | **.79** | .13 | .14 | -.01 | .09 | .28 | .29 | .01 |
| FFNI Manipulativeness | **.47** | .16 | **.76** | .14 | .10 | .11 | .18 | -.07 |
| FFNI Need for Admiration | **.35** | **.74** | .00 | .23 | -.05 | .02 | -.18 | -.22 |
| FFNI Reactive Anger | **.61** | **.52** | **.30** | .08 | .19 | .12 | -.02 | -.03 |
| FFNI Shame | .11 | **.86** | -.02 | -.02 | -.06 | .02 | -.18 | .03 |
| FFNI Thrill-Seeking | **.64** | .25 | **.33** | **.31** | .16 | -.01 | .26 | -.23 |
| NARQ Grandiosity | **.59** | .03 | **.38** | **.39** | **.33** | -.11 | .09 | .25 |
| NARQ Uniqueness | **.47** | .10 | **.41** | **.34** | **.33** | -.11 | .09 | **.48** |
| NARQ Charmingness | **.44** | .12 | **.66** | .19 | .21 | -.12 | .09 | .27 |
| NARQ Devaluation | **.76** | **.32** | .19 | .15 | -.03 | .15 | .12 | .09 |
| NARQ Supremacy | **.69** | **.30** | **.30** | .11 | .05 | .27 | .02 | .31 |
| NARQ Aggressiveness | **.68** | **.51** | **.30** | .13 | .09 | .05 | -.08 | .12 |
| PNI Contingent Self-Esteem | **.56** | **.69** | .20 | .26 | .07 | -.07 | -.08 | -.05 |
| PNI Exploitative | **.38** | .14 | **.79** | .22 | .12 | .02 | .18 | .07 |
| PNI Self-Sacrificing Self-Enhancement | .12 | **.50** | **.37** | **.32** | .26 | **-.37** | .15 | .29 |
| PNI Hiding the Self | .11 | **.80** | .08 | .01 | .01 | .21 | **.34** | .19 |
| PNI Grandiose Fantasy | .29 | .43 | **.35** | **.65** | .23 | .05 | .06 | .12 |
| PNI Devaluing | **.55** | **.66** | .19 | .24 | .10 | .08 | .08 | .03 |
| PNI Entitlement Rage | **.62** | **.56** | **.34** | .14 | .17 | .12 | -.01 | .09 |
| HSNS | .13 | -.02 | -.06 | -.02 | -.15 | **.91** | -.08 | .00 |

*Note:* Loadings ≥ . 30 in **boldface** font; ANT = antagonism, VLN = vulnerability, BLD = boldness, GRN = grandiosity; AS = attention-seeking, DIS = distrust, VLN = vulnerability, IND = indifference, UNQ = uniqueness.

**S10 Table**

*Incremental Effects of Dimensions Pathological Narcissism on Voting for Donald Trump in 2020*

|  | Echelon |  | OR | B | 95% HDI | *R*^2^ | Δ*R*^2^ |
| --- | --- | --- | --- | --- | --- | --- | --- |
| Step 1 |  | Age | .99 | -.01 | (-.02, .01) | .49* |  |
|  |  | Male | 1.15 | .14 | (-.29, .58) |  |  |
|  |  | White | 1.45 | .37 | (-.19, .94) |  |  |
|  |  | Heterosexual | .90 | -.11 | (-.70, .49) |  |  |
|  |  | College degree | .85 | -.16 | (-.63, .31) |  |  |
|  |  | Red State | .96 | -.04 | (-.48, .40) |  |  |
|  |  | GOP | 46.53* | 3.84 | (3.35, 4.33) |  |  |
| Step 2 | I | Age | .99 | -.01 | (-.01, .02) | .51* | .02 |
|  |  | Male | .93 | -.07 | (-.52, .38) |  |  |
|  |  | White | 1.45 | .37 | (-.21, .96) |  |  |
|  |  | Heterosexual | 1.23 | .21 | (-.44, .87) |  |  |
|  |  | College degree | .66 | -.41 | (-.88, .08) |  |  |
|  |  | Red State | 1.01 | .01 | (-.44, .46) |  |  |
|  |  | GOP | 42.52* | 3.75 | (3.25, 4.27) |  |  |
|  |  | Narcissism | 1.82* | .60 | (.34, .86) |  |  |
|  | II | Age | .99 | -.01 | (-.01, .02) | .51* | .02 |
|  |  | Male | .93 | -.07 | (-.53, .39) |  |  |
|  |  | White | 1.45 | .37 | (-.21, .97) |  |  |
|  |  | Heterosexual | 1.23 | .21 | (-.45, .88) |  |  |
|  |  | College degree | .66 | -.41 | (-.90, .09) |  |  |
|  |  | Red State | 1.01 | .01 | (-.45, .46) |  |  |
|  |  | GOP | 44.26* | 3.79 | (3.27, 4.29) |  |  |
|  |  | Grandiosity | 1.60* | .47 | (.22, .72) |  |  |
|  |  | Vulnerability | 1.45* | .37 | (.14, .61) |  |  |
|  | III | Age | .99 | -.01 | (-.01, .02) | .51* | .02 |
|  |  | Male | .89 | -.12 | (-.58, .34) |  |  |
|  |  | White | 1.49 | .40 | (-.19, 1.01) |  |  |
|  |  | Heterosexual | 1.21 | .19 | (-.46, .87) |  |  |
|  |  | College degree | .68 | -.38 | (-.87, .12) |  |  |
|  |  | Red State | 1.02 | .02 | (-.43, .47) |  |  |
|  |  | GOP | 43.38* | 3.77 | (3.28, 4.30) |  |  |
|  |  | Grandiosity | 1.58* | .46 | (.21, .70) |  |  |
|  |  | Vulnerability | 1.30* | .26 | (.04, .49) |  |  |
|  |  | Distrust | 1.38* | .32 | (.10, .54) |  |  |

*Note:* * indicates 0 is not within 95% HDI; HDI = highest density interval of parameter samples.

**S10 Table** (continued)

*Incremental Effects of Dimensions Pathological Narcissism on Voting for Donald Trump in 2020*

|  | Echelon |  | OR | B | 95% HDI | *R*^2^ | Δ*R*^2^ |
| --- | --- | --- | --- | --- | --- | --- | --- |
| Step 2 | IV | Age | 1.01 | .01 | (-.01, .02) | .51* | .02 |
|  |  | Male | .89 | -.12 | (-.59, .34) |  |  |
|  |  | White | 1.54 | .43 | (-.17, 1.05) |  |  |
|  |  | Heterosexual | 1.19 | .17 | (-.49, .86) |  |  |
|  |  | College degree | .64 | -.44 | (-.93, .06) |  |  |
|  |  | Red State | 1.02 | .02 | (-.44, .48) |  |  |
|  |  | GOP | 44.70* | 3.80 | (3.31, 4.32) |  |  |
|  |  | Antagonism | 1.77* | .57 | (.33, .82) |  |  |
|  |  | Grandiosity | 1.30* | .26 | (.03, .49) |  |  |
|  |  | Vulnerability | 1.14 | .13 | (-.10, .37) |  |  |
|  |  | Distrust | 1.08 | .08 | (-.15, .30) |  |  |
|  | V | Age | 1.00 | .00 | (-.02, .02) | .52* | .03 |
|  |  | Male | .83 | -.19 | (-.67, .28) |  |  |
|  |  | White | 1.45 | .37 | (-.24, 1.00) |  |  |
|  |  | Heterosexual | 1.35 | .30 | (-.38, 1.01) |  |  |
|  |  | College degree | .60* | -.51 | (-1.02, -.01) |  |  |
|  |  | Red State | .99 | -.01 | (-.47, .45) |  |  |
|  |  | GOP | 48.42* | 3.88 | (3.36, 4.42) |  |  |
|  |  | Antagonism | 1.93* | .66 | (.41, .91) |  |  |
|  |  | Grandiosity | 1.13 | .12 | (-.12, .36) |  |  |
|  |  | Vulnerability | 1.23 | .21 | (-.02, .44) |  |  |
|  |  | Distrust | .88 | -.13 | (-.36, .10) |  |  |
|  |  | Indifference | 1.43* | .36 | (.12, .59) |  |  |

*Note:* * indicates 0 is not within 95% HDI; HDI = highest density interval of parameter samples.

**S10 Table** (continued)

*Incremental Effects of Dimensions Pathological Narcissism on Voting for Donald Trump in 2020*

|  | Echelon |  | OR | B | 95% HDI | *R*^2^ | Δ*R*^2^ |
| --- | --- | --- | --- | --- | --- | --- | --- |
| Step 2 | VI | Age | 1.00 | .00 | (-.02, .02) | .52* | .03 |
|  |  | Male | .83 | -.19 | (-.67, .28) |  |  |
|  |  | White | 1.49 | .40 | (-.23, 1.04) |  |  |
|  |  | Heterosexual | 1.32 | .28 | (-.42, .99) |  |  |
|  |  | College degree | .59* | -.52 | (-1.03, -.02) |  |  |
|  |  | Red State | 1.00 | .00 | (-.46, .46) |  |  |
|  |  | GOP | 47.94* | 3.87 | (3.35, 4.41) |  |  |
|  |  | Antagonism | 1.90* | .64 | (.39, .90) |  |  |
|  |  | Boldness | 1.28* | .25 | (.01, .49) |  |  |
|  |  | Vulnerability | 1.23 | .21 | (-.02, .44) |  |  |
|  |  | Grandiosity | .93 | -.07 | (-.31, .18) |  |  |
|  |  | Distrust | .87 | -.14 | (-.37, .09) |  |  |
|  |  | Indifference | 1.39* | .33 | (.09, .56) |  |  |
|  | VII | Age | 1.00 | .00 | (-.02, .02) | .52* | .03 |
|  |  | Male | .83 | -.19 | (-.67, .28) |  |  |
|  |  | White | 1.49 | .40 | (-.23, 1.04) |  |  |
|  |  | Heterosexual | 1.32 | .28 | (-.42, 1.00) |  |  |
|  |  | College degree | .59* | -.52 | (-1.03, -.01) |  |  |
|  |  | Red State | 1.00 | .00 | (-.47, .46) |  |  |
|  |  | GOP | 48.42* | 3.88 | (3.37, 4.43) |  |  |
|  |  | Antagonism | 1.92* | .65 | (.40, .90) |  |  |
|  |  | Vulnerability | 1.23 | .21 | (-.02, .45) |  |  |
|  |  | Boldness | 1.27* | .24 | (.00, .48) |  |  |
|  |  | Attention-Seeking | .99 | -.01 | (-.24, .23) |  |  |
|  |  | Distrust | .87 | -.14 | (-.37, .09) |  |  |
|  |  | Grandiosity | .90 | -.10 | (-.34, .14) |  |  |
|  |  | Indifference | 1.38* | .32 | (.09, .56) |  |  |

*Note:* * indicates 0 is not within 95% HDI; HDI = highest density interval of parameter samples.

**S10 Table** (continued)

*Incremental Effects of Dimensions Pathological Narcissism on Voting for Donald Trump in 2020*

|  | Echelon |  | OR | B | 95% HDI | *R*^2^ | Δ*R*^2^ |
| --- | --- | --- | --- | --- | --- | --- | --- |
| Step 2 | VIII | Age | 1.00 | .00 | (-.02, .02) | .52* | .03 |
|  |  | Male | .83 | -.19 | (-.67, .29) |  |  |
|  |  | White | 1.49 | .40 | (-.21, 1.04) |  |  |
|  |  | Heterosexual | 1.32 | .28 | (-.42, 1.00) |  |  |
|  |  | College degree | .59* | -.52 | (-1.03, -.01) |  |  |
|  |  | Red State | 1.00 | .00 | (-.46, .46) |  |  |
|  |  | GOP | 48.91* | 3.89 | (3.37, 4.44) |  |  |
|  |  | Antagonism | 1.92* | .65 | (.40, .91) |  |  |
|  |  | Vulnerability | 1.25 | .22 | (-.02, .45) |  |  |
|  |  | Boldness | 1.28* | .25 | (.01, .49) |  |  |
|  |  | Grandiosity | .90 | -.10 | (-.34, .13) |  |  |
|  |  | Attention-Seeking | 1.00 | .00 | (-.24, .24) |  |  |
|  |  | Distrust | .88 | -.13 | (-.36, .09) |  |  |
|  |  | Indifference | 1.35* | .30 | (.07, .54) |  |  |
|  |  | Uniqueness | 1.01 | .01 | (-.22, .24) |  |  |

*Note:* * indicates 0 is not within 95% HDI; HDI = highest density interval of parameter samples.

**S11 Table**

*Eigenvalues, Variance Accounted for, and Loadings for* *EFA for the FFNI*

|  | E-E | Auth | Shame | A-S | Distrust | T-S |
| --- | --- | --- | --- | --- | --- | --- |
| Eigenvalue | 23.62 | 6.23 | 3.73 | 2.19 | 1.75 | 1.53 |
| % of variance | 39.37 | 10.38 | 6.22 | 3.65 | 2.92 | 2.55 |
| I am extremely ambitious | -.02 | .11 | -.06 | **.70** | .03 | .13 |
| Others say I brag too much, but everything I say is true | **.74** | .03 | .05 | .01 | -.07 | .09 |
| Leadership comes easy for me | -.05 | **.76** | -.03 | .23 | .02 | -.13 |
| When someone does something nice for me, I wonder what they want from me | **.37** | -.12 | .29 | .11 | **.39** | .05 |
| I deserve to receive special treatment | **.97** | -.04 | -.03 | .08 | -.21 | -.11 |
| I get lots of enjoyment from entertaining others | -.07 | **.48** | -.04 | .06 | -.32 | .28 |
| It's fine to take advantage of persons to get ahead | **.86** | -.01 | -.07 | -.09 | -.05 | .08 |
| I often fantasize about someday being famous | **.49** | .12 | .12 | .05 | -.11 | .16 |
| When people judge me, I just don't care | .12 | .23 | **-.40** | .03 | .37 | .23 |
| I don't worry about others' needs | **.76** | -.09 | .02 | -.01 | .27 | -.08 |
| I'm pretty good at manipulating people | .29 | **.56** | .12 | -.27 | .11 | .12 |
| I often feel as if I need compliments from others in order to be sure of myself | **.45** | -.03 | **.41** | .04 | -.10 | .13 |
| I hate being criticized so much that I can't control my temper when it happens | **.59** | .05 | **.35** | -.01 | .08 | -.06 |
| When I realize I have failed at something, I feel humiliated | .03 | .11 | **.76** | .00 | .15 | -.06 |
| I will try almost anything to get my "thrills" | **.47** | -.06 | .01 | .03 | .04 | **.50** |
| I have a tremendous drive to succeed | -.03 | .16 | .01 | **.81** | .11 | .02 |
| I only associate with people of my caliber | **.77** | .09 | .06 | .04 | .05 | -.11 |
| I am comfortable taking on positions of authority | -.16 | **.81** | -.09 | .22 | .01 | -.12 |
| I trust that other people will be honest with me | **.33** | .12 | -.18 | .04 | **-.48** | -.03 |
| I don't think the rules apply to me as much as they apply to others | **.88** | -.12 | -.03 | -.01 | -.11 | .10 |

*Note*. Six eigenvalues (23.62, 6.23, 3.73, 2.19, 1.75, 1.53) exceeded the random values from a parallel analysis whereas the seventh eigenvalue (1.32) was below that suggested from a parallel analysis (1.49); factors loadings ≥ |.30| indicated by **boldfaced** font.

**S11 Table** (continued)

*Eigenvalues, Variance Accounted for, and Loadings for* *EFA for the FFNI*

|  | E-E | Auth | Shame | A-S | Distrust | T-S |
| --- | --- | --- | --- | --- | --- | --- |
| I like being noticed by others | .31 | **.39** | .10 | .03 | -.28 | .08 |
| I will use persons as tools to advance myself | **.82** | .14 | -.01 | -.11 | -.05 | -.02 |
| I often fantasize about having lots of success and power | **.52** | .22 | .13 | .08 | -.11 | .03 |
| I don't really care what others think of me | .17 | .19 | **-.39** | .01 | **.41** | .11 |
| I don't generally pay much attention to the woes of others | **.74** | -.14 | -.13 | -.03 | **.33** | -.04 |
| I can maneuver people into doing things | .24 | **.61** | .12 | -.18 | .12 | .07 |
| I am stable in my sense of self | -.05 | .33 | -.43 | .04 | .00 | -.12 |
| I have at times gone into a rage when not treated rightly | **.38** | .15 | .23 | -.03 | .12 | .17 |
| I feel awful when I get put down in front of others | -.11 | .07 | **.69** | -.04 | .09 | -.06 |
| I am a bit of a daredevil | .19 | -.01 | -.02 | .11 | .16 | .65 |
| I aspire for greatness | .05 | .15 | .05 | **.59** | -.05 | .16 |
| I do not waste my time hanging out with people who are beneath me | **.75** | .03 | .05 | .00 | .03 | -.06 |
| Persons generally follow my lead and authority | .03 | **.78** | -.05 | .21 | -.03 | -.21 |
| I'm slow to trust people | .01 | -.11 | .28 | .11 | **.58** | .08 |
| It may seem unfair, but I deserve extra (i.e., attention, privileges, rewards) | **.95** | -.15 | -.07 | .10 | -.15 | .03 |
| I like being the most popular person at a party | **.48** | **.43** | .02 | -.04 | -.26 | .05 |
| Sometimes to succeed you need to use other people | **.78** | .07 | -.05 | -.12 | -.02 | .07 |
| I rarely fantasize about becoming famously successful | -.05 | .09 | .01 | -.22 | .17 | -.05 |
| I'm pretty indifferent to the criticism of others | **.39** | .11 | **-.45** | .01 | .20 | .12 |
| I'm not big on feelings of sympathy | **.80** | -.15 | -.09 | -.04 | .33 | -.05 |
| I can talk my way into and out of anything | .15 | **.61** | .00 | -.04 | .08 | .14 |
| I feel very insecure about whether I will achieve much in life | .23 | -.15 | **.55** | -.02 | .17 | .15 |

**S11 Table** (continued)

*Eigenvalues, Variance Accounted for, and Loadings for* *EFA for the FFNI*

|  | E-E | Auth | Shame | A-S | Distrust | T-S |
| --- | --- | --- | --- | --- | --- | --- |
| It really makes me angry when I don't get what I deserve | .25 | .31 | **.42** | .00 | .17 | -.03 |
| I feel ashamed when people judge me | .09 | -.04 | **.77** | .00 | .00 | .02 |
| I would risk injury to do something exciting | **.37** | -.13 | -.03 | .08 | .15 | **.63** |
| I am driven to succeed | -.02 | .11 | -.02 | **.80** | .08 | .07 |
| I am a superior person | **.77** | .09 | -.13 | .22 | -.07 | -.11 |
| I tend to take charge of most situations | -.02 | **.72** | -.02 | .28 | .05 | -.11 |
| I often think that others aren't telling me the whole truth | .10 | -.01 | **.36** | .10 | **.48** | .14 |
| I believe I am entitled to special accommodations | **.94** | -.06 | -.03 | .08 | -.18 | -.03 |
| I love to entertain people | -.02 | **.50** | -.03 | .03 | **-.30** | .30 |
| I'm willing to exploit others to further my own goals | **.80** | .07 | .00 | -.09 | -.03 | .08 |
| Someday I believe that most people will know my name | **.69** | .12 | -.06 | .11 | -.15 | .06 |
| Others' opinions of me are of little concern to me | .19 | .22 | **-.30** | -.08 | **.44** | .12 |
| I don't get upset by the suffering of others | **.86** | -.09 | -.07 | -.06 | .27 | -.16 |
| It is easy to get people to do what I want | .27 | **.71** | -.01 | -.20 | -.01 | .01 |
| I wish I didn't care so much about what others think of me | .11 | -.08 | **.70** | .03 | -.04 | .03 |
| I feel enraged when people disrespect me | .27 | .21 | **.49** | .07 | .18 | -.04 |
| I feel foolish when I make a mistake in front of others | -.13 | .07 | **.82** | -.03 | .20 | -.12 |
| I like doing things that are risky or dangerous | .27 | -.08 | -.04 | .09 | .12 | **.72** |
|  | Factor Correlations | | | | | |
| Entitled Exploitativeness | — |  |  |  |  |  |
| Authortativeness | .63 | — |  |  |  |  |
| Shame | .30 | .00 | — |  |  |  |
| Acclaim-Seeking | .31 | .48 | -.03 | — |  |  |
| Distrust | .32 | .17 | -.15 | -.04 | — |  |
| Thrill-Seeking | .65 | .55 | .27 | .24 | .05 | — |

**S12 Table**

*Eigenvalues, Variance Accounted for, and Loadings for* *EFA for the PNI*

|  | Vuln | G-F | Exploit | Hiding | SSSE |
| --- | --- | --- | --- | --- | --- |
| Eigenvalue | 24.00 | 3.55 | 2.44 | 1.87 | 1.57 |
| % of variance | 46.16 | 6.83 | 4.69 | 3.60 | 3.02 |
| I often fantasize about being admired and respected. | .15 | **.74** | .08 | -.14 | -.05 |
| My self-esteem fluctuates a lot. | **.58** | **.31** | -.14 | .02 | -.17 |
| I sometimes feel ashamed about my expectations of others when they disappoint me. | **.58** | .15 | -.05 | .17 | -.01 |
| I can usually talk my way out of anything. | -.10 | .13 | **.86** | -.01 | -.07 |
| It’s hard for me to feel good about myself when I’m alone. | **.75** | .08 | -.05 | -.02 | -.06 |
| I can make myself feel good by caring for others. | -.03 | -.17 | -.04 | -.01 | **.64** |
| I hate asking for help. | -.14 | .02 | -.07 | **.68** | .02 |
| When people don’t notice me, I start to feel bad about myself. | **.98** | .00 | -.07 | -.13 | -.04 |
| I often hide my needs for fear that others will see me as needy and dependent. | **.38** | .07 | -.10 | **.54** | -.12 |
| I can make anyone believe anything I want them to. | .01 | .11 | **.87** | -.04 | -.09 |
| I get mad when people don’t notice all that I do for them. | **.51** | .02 | .26 | .16 | -.04 |
| I get annoyed by people who are not interested in what I say or do. | **.69** | -.12 | .20 | .08 | .01 |
| I wouldn’t disclose all my intimate thoughts and feelings to someone I didn’t admire. | -.23 | -.04 | .08 | **.52** | .18 |
| I often fantasize about having a huge impact on the world around me. | .04 | **.81** | .07 | -.06 | -.06 |
| I find it easy to manipulate people. | .22 | .00 | **.80** | -.05 | -.15 |
| When others don’t notice me, I start to feel worthless. | **.98** | -.02 | -.10 | -.06 | .01 |
| Sometimes I avoid people because I’m concerned that they’ll disappoint me. | **.54** | .06 | .01 | .28 | -.10 |
| I typically get very angry when I’m unable to get what I want from others. | **.78** | -.13 | .29 | -.03 | -.06 |
| I sometimes need important others in my life to reassure me of my self-worth. | **.82** | .08 | -.13 | -.11 | .11 |

*Note*. Five eigenvalues (24.00, 3.56, 2.44, 1.87, 1.57) exceeded the random values from a parallel analysis whereas the sixth eigenvalue (1.13) was below that suggested from a parallel analysis (1.46); factors loadings ≥ |.30| indicated by **boldfaced** font.

**S12** **Table** (continued)

*Eigenvalues, Variance Accounted for, and Loadings for* *EFA for the PNI*

|  | Vuln | G-F | Exploit | Hiding | SSSE |
| --- | --- | --- | --- | --- | --- |
| When I do things for other people, I expect them to do things for me. | **.50** | -.08 | **.34** | .08 | .02 |
| When others don’t meet my expectations, I often feel ashamed about what I wanted. | **.70** | .07 | -.03 | .08 | .02 |
| I feel important when others rely on me. | -.08 | .23 | .07 | .15 | **.50** |
| I can read people like a book. | -.19 | .03 | **.60** | .08 | .20 |
| When others disappoint me, I often get angry at myself. | **.69** | .11 | .01 | .02 | -.02 |
| Sacrificing for others makes me the better person. | -.02 | .00 | .00 | .11 | **.60** |
| I often fantasize about accomplishing things that are probably beyond my means. | -.08 | **.86** | .03 | .07 | -.08 |
| Sometimes I avoid people because I’m afraid they won’t do what I want them to do. | **.75** | -.08 | .04 | .13 | -.01 |
| It’s hard to show others the weaknesses I feel inside. | .13 | -.08 | -.11 | **.71** | .03 |
| I get angry when criticized. | **.59** | -.16 | .10 | .23 | .01 |
| It’s hard to feel good about myself unless I know other people admire me. | **.90** | .02 | -.01 | -.10 | .07 |
| I often fantasize about being rewarded for my efforts. | .02 | **.72** | .04 | .08 | .05 |
| I am preoccupied with thoughts and concerns that most people are not interested in me. | **.77** | .18 | -.08 | -.10 | .00 |
| I like to have friends who rely on me because it makes me feel important. | **.32** | .14 | .07 | -.02 | **.41** |
| Sometimes I avoid people because I’m concerned they won’t acknowledge what I do for them. | **.80** | -.06 | .10 | -.03 | .03 |
| Everybody likes to hear my stories. | .02 | .02 | **.56** | -.14 | .27 |
| It’s hard for me to feel good about myself unless I know other people like me. | **.94** | .02 | -.12 | -.12 | .04 |
| It irritates me when people don’t notice how good a person I am. | **.81** | -.10 | .12 | -.01 | .06 |
| I will never be satisfied until I get all that I deserve. | **.39** | .12 | **.38** | -.03 | .04 |
| I try to show what a good person I am through my sacrifices. | .13 | .08 | -.01 | .06 | **.60** |

**S12** **Table** (continued)

*Eigenvalues, Variance Accounted for, and Loadings for* *EFA for the PNI*

|  | Vuln | G-F | Exploit | Hiding | SSSE |
| --- | --- | --- | --- | --- | --- |
| I am disappointed when people don’t notice me. | **.89** | -.02 | -.05 | -.13 | .09 |
| I often find myself envying others’ accomplishments. | **.48** | .22 | -.06 | .15 | .01 |
| I often fantasize about performing heroic deeds. | .16 | **.76** | .06 | -.10 | -.04 |
| I help others in order to prove I’m a good person. | **.36** | .05 | -.10 | -.01 | **.55** |
| It’s important to show people I can do it on my own even if I have some doubts inside. | -.19 | .06 | .06 | **.48** | **.36** |
| I often fantasize about being recognized for my accomplishments. | .08 | **.76** | .05 | -.01 | .02 |
| I can’t stand relying on other people because it makes me feel weak. | .16 | -.08 | .08 | **.68** | -.04 |
| When others don’t respond to me the way that I would like them to, it is hard for me to still feel ok with myself. | **.92** | -.08 | -.06 | .01 | .05 |
| I need others to acknowledge me. | **.92** | .02 | -.04 | -.21 | .07 |
| I want to amount to something in the eyes of the world. | -.04 | **.57** | .06 | .06 | .18 |
| When others get a glimpse of my needs, I feel anxious and ashamed. | **.51** | .09 | -.17 | **.46** | -.10 |
| Sometimes it’s easier to be alone than to face not getting everything I want from other people. | **.35** | .10 | .03 | **.40** | -.02 |
| I can get pretty angry when others disagree with me. | **.70** | -.12 | .17 | .09 | -.05 |
|  | Factor Correlations | | | | |
| Vulnerability | — |  |  |  |  |
| Grandiose Fantasy | .69 | — |  |  |  |
| Exploitation | .57 | .58 | — |  |  |
| Hiding the Self | .55 | .42 | .30 | — |  |
| Self-Sacrificing Self-Enhancement | .43 | .59 | .50 | .19 | — |

**S13 Table**

*Eigenvalues, Variance Accounted for, and Loadings for* *EFA for the NARQ*

|  | Rivalry | Admiration |
| --- | --- | --- |
| Eigenvalue | 9.62 | 1.92 |
| % of variance | 53.47 | 10.68 |
| I am great | **-.31** | **.97** |
| I will someday be famous | .20 | **.66** |
| I show others how special I am | .00 | **.85** |
| I react annoyed if another person steals the show from me | **.67** | .20 |
| I enjoy my successes very much | -.12 | **.46** |
| I secretly take pleasure in the failure of my rivals | **.78** | .02 |
| Most of the time I am able to draw people's attention to myself in conversations | .17 | **.59** |
| I deserve to be seen as a great personality | .12 | **.78** |
| I want my rivals to fail | **.82** | -.08 |
| I enjoy it when another person is inferior to me | **.79** | .11 |
| I often get annoyed when I am criticized | **.69** | -.16 |
| I can barely stand it if another person is at the center of events | **.70** | .23 |
| Most people won't achieve anything | **.87** | -.20 |
| Other people are worth nothing | **.79** | -.04 |
| Being a very special person gives me a lot of strength | .14 | **.72** |
| I manage to be the center of attention with my outstanding contributions | .27 | **.65** |
| Most people are somehow losers | **.90** | -.06 |
| Mostly, I am very adept at dealing with other people | -.13 | **.61** |
|  | Factor Correlations | |
| Rivalry | — |  |
| Admiration | .71 | — |

*Note*. Two eigenvalues (9.62, 1.92) exceeded the random values from a parallel analysis whereas the third eigenvalue (1.11) was below that suggested from a parallel analysis (1.23); factors loadings ≥ |.30| indicated by **boldfaced** font.

**S14 Table**

*Eigenvalues, Variance Accounted for, and Loadings for* *EFA for the HSNS*

|  | S-Con | S-Cen |
| --- | --- | --- |
| Eigenvalue | 4.22 | 1.68 |
| % of variance | 42.16 | 16.77 |
| I can become entirely absorbed in thinking about my personal affairs, my health, my cares or my relations to others. | **.83** | .03 |
| My feelings are easily hurt by ridicule or the slighting remarks of others. | **.88** | -.18 |
| When I enter a room I often become self-conscious and feel that the eyes of others are upon me. | **.86** | -.06 |
| I dislike sharing the credit of an achievement with others. | **.71** | -.09 |
| I feel that I have enough on my hands without worrying about other people's troubles. | **.57** | .10 |
| I feel that I am temperamentally different from most people. | .29 | **.53** |
| I often interpret the remarks of others in a personal way. | .24 | **.55** |
| I easily become wrapped up in my own interests and forget the existence of others. | .00 | **.72** |
| I dislike being with a group unless I know that I am appreciated by at least one of those present. | -.18 | **.57** |
| I am secretly "put out" or annoyed when other people come to me with their troubles, asking me for my time and sympathy. | -.14 | **.46** |
|  | Factor Correlations | |
| Self-conscious | — |  |
| Self-centered | .51 | — |

*Note*. Two eigenvalues (4.22, 1.68) exceeded the random values from a parallel analysis whereas the third eigenvalue (.83) was below that suggested from a parallel analysis (1.20); factors loadings ≥ |.30| indicated by **boldfaced** font.
